# Initial Surgical Results of Subcostal Single‐Port Robotic Lung Resection: A Comparative Analysis With the Multi‐Port Robot

**DOI:** 10.1002/wjs.70387

**Published:** 2026-05-14

**Authors:** Teruhisa Kawaguchi, Fumiaki Watanabe, Shinji Kaneda, Daisuke Ito, Koji Kawaguchi

**Affiliations:** ^1^ Department of Thoracic Surgery Mie University Tsu Mie Japan

**Keywords:** da Vinci SP system, minimally invasive thoracic surgery, single‐port robotic surgery

## Abstract

**Background:**

The da Vinci SP enables single‐port robot‐assisted thoracoscopic surgery and this modality represents a novel approach to lung resection via the subcostal arch technique. We implemented this technique at our institution, performed 30 surgeries, and then compared the outcomes with those of surgeries using the da Vinci Xi system.

**Methods:**

Thirty consecutive patients in each group with stage I–III lung cancer, who were all operated on with da Vinci SP and Xi, were compared.

**Results:**

The SP group included 24 lobectomies and 6 segmentectomies. The Xi group included 16 lobectomies, 1 bilobectomy, and 13 segmentectomies. No conversion was required in either group. An additional port was needed in two cases in the SP group. There were no significant differences in the perioperative outcomes between the SP and Xi groups. Postoperative complications occurred in two patients in the SP group and nine patients in the Xi group. No complications classified as Clavien‐Dindo grade ≥ 3 were observed in the SP group.

**Conclusions:**

Anatomic lung resection using the da Vinci SP system demonstrated outcomes that were comparable to those of the da Vinci Xi system.

**Trial Registration:**

Clinical registration number: approval number, H2025‐092; date of approval, 2025.6.3, Mie university Hospital

## Graphical abstract

This study demonstrated anatomic lung resection using the da Vinci SP, a novel single‐port robot‐assisted thoracoscopic approach, yields outcomes comparable to those achieved with the da Vinci Xi.

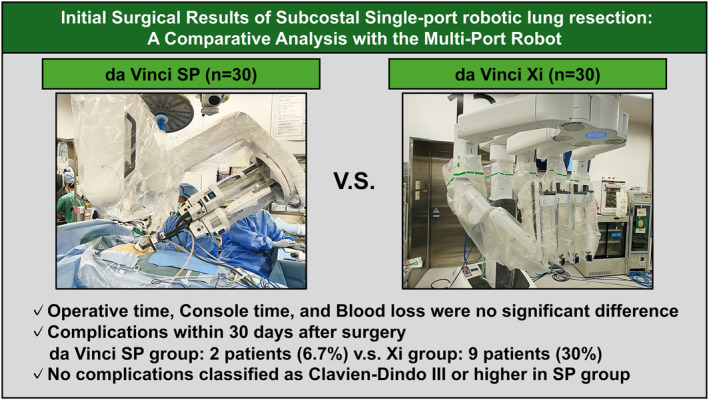

## 1 Introduction

Minimally invasive surgery in the field of thoracic surgery began in the 1990s with thoracoscopic surgery [1]. Currently, multi‐port robotic surgery and single port thoracoscopic surgery are established approaches [2]. Additionally, innovations in single‐incision surgery utilizing multi‐arm robotic systems have also been reported [3].

The da Vinci SP System, developed by Intuitive Surgical, is a single‐port robotic platform approved since September 2022 in Japan, enabling robotic surgery through a single incision with a single articulated arm. Our hospital traditionally performs robotic lung resections using the da Vinci Xi system. However, in February 2024, we started to perform anatomical lung resections via a subcostal approach using the da Vinci SP system.

To date, we have performed 30 lung resections for lung cancer, using the da Vinci SP system. In this study, we compared the initial outcomes of da Vinci SP at our hospital with the surgical results of da Vinci Xi performed during the same period and examined its short‐term outcomes and safety.

## 2 Material and Methods

This study was conducted in accordance with the Declaration of Helsinki and was approved by the Clinical Research Ethics Review Committee of Mie University Hospital (approval number, H2025‐092; date of approval, 2025.6.3). All surgeries and data collection were performed at Mie University Hospital, Tsu, Japan. The clinical outcome data were obtained from hospital records. All methods were performed in accordance with the relevant guidelines and regulations.

Between February and November 2024, anatomical lung resections using the da Vinci SP or da Vinci Xi systems were performed in patients with lung cancer at Mie University Hospital. The indication criteria for robot‐assisted thoracic surgery (RATS) using both da Vinci systems were as follows: stage I to III lung cancer without bulky lymph node (defined as ≥ 3 cm) metastasis, and no requirement for angioplasty or pneumonectomy. Lower lobectomies were performed for the initial da Vinci SP cases. After gaining experience with bilateral lower lobectomies, the indication was expanded to include anatomical lung resections without clear adhesions or lymph node metastasis. Once all types of lobectomies were performed, the indication was further broadened to include anatomical lung resections that did not require extended surgery. Chest wall resection concomitant with lobectomy was indicated only for da Vinci Xi [4]. A total of 60 patients were included, comprising 30 consecutive patients in the da Vinci SP group and 30 in the da Vinci Xi group. The da Vinci Xi procedures included in this study were performed during the same period as the da Vinci SP procedures by surgeons who had already completed their learning curve for the da Vinci Xi procedure, allowing for a comparison of the initial da Vinci SP outcomes with those for the established procedure using the da Vinci Xi. The procedures were performed by one primary surgeon for the da Vinci SP system (27 years of clinical experience) and by two primary surgeons for the da Vinci Xi system (27 and 13 years of clinical experience, respectively). The study evaluated the operative time, console time (from the start to the completion of console manipulation), blood loss, number of lymph nodes removed, drainage duration, postoperative hospital stay, and postoperative complications within 30 days. Postoperative complications were assessed using the Clavien‐Dindo classification [5].

### 2.1 Setting Up the da Vinci SP

The patient was positioned in the lateral recumbent position, with the operating table in a 10‐degree mountain fold. A 4‐cm skin incision was made along the subcostal arch, one fingerbreadth ventral to the eighth rib. After making a partial incision in the rectus abdominis muscle, the dissection was advanced ventrally to expose the peritoneum. A partial incision was made in the diaphragm to access the thoracic cavity. The diaphragm and subcutaneous tissues were secured by ligation. An Access Port Kit Large (Intuitive Surgical) was inserted for the da Vinci SP, and CO_2_ insufflation was initiated at a pressure of 8 mmHg using the AirSeal system (ConMed, USA) (Video 1). A Cadiere Forceps (1st arm), Maryland Bipolar Forceps (2nd arm) and Round Tooth Retractor (3rd arm) (Intuitive Surgical, Sunnyvale, CA, USA) were used, as shown in Video 1.

**Video 1:** Right middle lobectomy for Stage I lung cancer using the da Vinci SP.

### 2.2 The Setting of da Vinci Xi

The patient was positioned in the lateral recumbent position, with the operating table in a 10‐degree mountain fold. Our preferred port placement consists of four ports in the eighth or ninth intercostal spaces and another in the fifth site, as previously described [6]. 8 mmHg of CO_2_ insufflation is performed using the AirSeal system.

### 2.3 Statistical Analysis

For continuous variables, the normality of the data was assessed using the Shapiro‐Wilk test, and the unpaired *t*‐test was applied accordingly. Categorical variables were analyzed using Fisher's exact test. In all statistical tests, differences between groups were considered to be significant at *p* < 0.05. All statistical analyses were performed using EZR (Saitama Medical Center, Jichi Medical University, Saitama, Japan), a graphical user interface for R (R Foundation for Statistical Computing, Vienna, Austria).

## 3 Results

**FIGURE 1:**
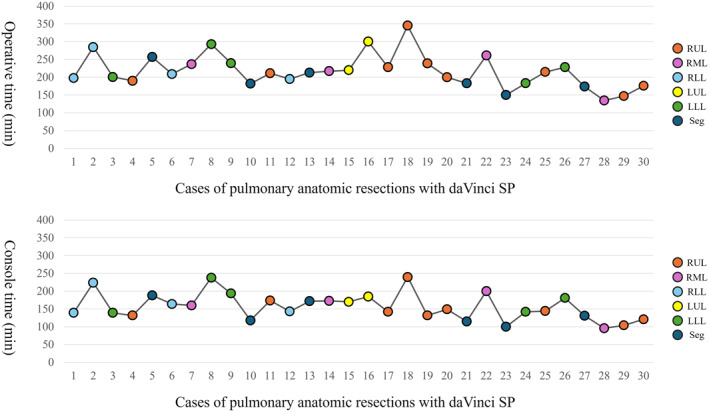
The operative and console times for lung resection in the da Vinci SP group are presented in the order of surgeon experience. LLL, left lower lobectomy; LUL, left upper lobectomy; RLL, right lower lobectomy; RML right middle lobectomy; RUL, right upper lobectomy; Seg, segmentectomy.

Sixty patients who had undergone anatomical lung resection were enrolled in this study. Among them, 31 were males and 29 were females. Of these, 56 patients had primary lung cancer, and 4 cases involved metastatic lung cancer, with no significant differences in the patient characteristics (Table 1). Lobectomy was performed in 24 patients in the da Vinci SP group and in 16 patients in the da Vinci Xi group (Table 2). Segmentectomy was performed in six patients in the SP group and 13 in the Xi group. One patient in the Xi group underwent a more complex procedure that involved simultaneous bilobectomy and bronchoplasty. Another patient in the Xi group underwent right upper lobectomy with a combined resection of the vertebral body and fourth and fifth ribs. No significant differences were found between the two groups in terms of operative time, console time, or intraoperative blood loss. No conversions were observed in either group. In the SP group, two patients required the addition of one port. One case involved a left upper lobectomy in which an additional port was placed in the 5th intercostal space for the processing of A1+2a + A3. The other case was a left S1 + 2 segmentectomy, in which an additional port was required in the 6th intercostal space because the SP instruments could not reach the pulmonary apex during adhesion dissection. The operative and console times for lung resection in the da Vinci SP group are presented in the order of surgical experience (Figure 1). The longest operative time occurred during the right upper lobectomy in Case 15, which also involved diagnostic segmentectomy. After Case 23, the operative time never exceeded 4 h, and all subsequent cases were completed using the da Vinci SP without the need for an additional port.

**TABLE 1:** Comparison of the patient characteristics between the SP and Xi groups.

Variable	SP	Xi	*p* value
Age (years)	69.9 ± 11.8	70.6 ± 8.8	0.796
Sex (men/women)			1.000
Men	16	15	
Women	14	15	
Body mass index (kg/m^2^)	22.5 ± 3.7	22.3 ± 4.0	0.795
Brinkman index	416 ± 632	364 ± 510	0.726
Preoperative ECOG performance status			0.612
0	29	27	
1	1	3	
Preoperative pulmonary function test			
%VC (%)	99.4 ± 11.3	98.6 ± 13.8	0.814
FEV1% (%)	76.5 ± 9.6	76.5 ± 6.2	0.992
Tumor size (cm)	2.5 ± 1.2	2.8 ± 1.2	0.387
Solid size (cm)	2.0 ± 1.2	1.8 ± 1.5	0.567
Clinical TNM			
T1	22	19	0.547
T2	3	6	0.469
T3	2	0	0.491
T4	0	1	1.000
N0	27	24	0.352
N1	1	2	1.000
N2	0	2	0.491
Clinical stage			
Stage I	23	19	0.355
Stage II	4	5	1.000
Stage III	0	2	0.491

**TABLE 2:** Comparison of the perioperative factors between the SP and Xi groups.

Variable	SP	Xi	*p* value
Lobectomy	24	16	0.054
Right upper lobe	9	6	0.552
Right middle lobe	4	2	0.671
Right lower lobe	4	3	1.000
Left upper lobe	2	2	1.000
Left lower lobe	5	3	0.706
Bilobectomy	0	1	1.000
Right middle and lower lobe	0	1	1.000
Segmentectomy	6	13	0.095
Right S1	1	1	1.000
Right S3	0	1	1.000
Right S6	2	0	0.492
Left S1 + 2	1	5	0.195
Left S1 + 2 + 3	0	2	0.492
Left S3 + 4 + 4	0	1	1.000
Left S6	2	1	1.000
Left S8 + 9	0	1	1.000
Left S9 + 10	0	1	1.000
Operative time (min)	217.1 ± 47.1	220.9 ± 62.2	0.789
Console time (min)	157.0 ± 38.2	166.5 ± 57.1	0.452
A amount of bleeding (mL)	21.2 ± 41.7	17.2 ± 48.2	0.734
Total number of stapler	6.3 ± 2.5	6.9 ± 2.0	0.313
Transfusion	0	0	—
Conversion	0	0	—
Total number of lymph nodes harvested	11.5 ± 7.8	11.6 ± 10.4	0.961
Histological type			
Adenocarinoma	23	26	0.506
Squamous cell carcinoma	5	1	0.195
Metastatic lung cancer	2	2	1.000
Other	0	1	1.000
Pathological stage			
0	1	5	0.195
I	23	15	0.060
II	1	5	0.195
III	2	3	1.000
IV	1	0	1.000

There were no significant differences in the duration of postoperative drainage or hospital stay, and all patients were discharged (Table 3). Complications within 30 days after surgery occurred in 2 patients (6.7%) in the da Vinci SP group and 9 patients (30%) in the da Vinci Xi group. However, there was no significant difference in the incidence of complications classified as Clavien‐Dindo grade II or higher (*p* = 0.080). Four complications, classified as Clavien‐Dindo grade III or higher, occurred in the Xi group. Pyothorax occurred in the patients who underwent bilobectomy and bronchoplasty. Pneumothorax caused by midlobar torsion occurred after right upper lobectomy. Bronchopleural fistula occurred in two cases: one after resection of the right S3 area, requiring pleurodesis with OK‐432, and the other after left upper lobectomy, which required pulmonary suturing.

**TABLE 3:** Comparison of the short‐term postoperative outcomes between the SP and Xi groups.

Variable	SP	Xi	*p* value
Chest tube duration (days)	2.3 ± 0.9	3.3 ± 3.6	0.156
Postoperative hospital stays (days)	5.7 ± 2.7	8.6 ± 8.1	0.068
Postoperative complications within 30 days (Clavien–Dindo classification)			
None	28	21	0.042
I	0	1	
Surgical site infection	0	1	
II	2	4	
Pneumonia	1	2	
Reflux esophagitis	1	0	
Others	0	2	
IIIa	0	1	
Prolonged leakage	0	1	
IIIb	0	3	
Empyema	0	1	
Prolonged leakage	0	1	
Atelectasis	0	1	

## 4 Discussion

Lung cancer surgery using the da Vinci surgical robot was first reported in 2002 [7]. Since then, robotic platforms have evolved significantly, leading to the report by Gonzalez‐Rivas et al. in 2022 regarding lung resection through a single intercostal space using the da Vinci Xi system [3]. The latest fourth‐generation model, the da Vinci SP, integrates three surgical forceps and a camera into a single robotic arm, thus enabling single‐incision robotic surgery. However, because a minimum port length of 2.5 cm is required for a single wound, surgery via the intercostal route is not recommended in Japan. To address this problem, we introduced the subcostal arch approach [8, 9]. Our experience aligns with the findings of Bulgarelli et al. and Lee et al., who demonstrated that the subcostal approach can be performed safely with minimal high‐grade complications (Clavien‐Dindo Grade ≥ III) [10, 11].

The basic console operation of the da Vinci SP is the same as that of the da Vinci Xi. Although the da Vinci SP has only one arm, its flexible graspers and camera allow for reduced instrument fighting. However, the current SP lacks energy devices, staplers, and suction instruments, the assistant must perform these roles [11, 12]. At our hospital, to minimize instrument fighting, we used long‐handled curved instruments similar to those used in single‐port video‐assisted thoracic surgery (VATS). In this study, as there were no significant differences in surgical time, console time, blood loss, and the number of lymph nodes dissected, we suggest that essential oncological procedures can be performed with da Vinci SP as safely as with Xi.

Additionally, in the da Vinci SP surgery cases, 28 cases (93.3%) were completed using a single‐port, while two patients required the addition of a port. In one case, an additional port was required to remove adhesions at the pulmonary apex, as the da Vinci SP arm was not physically accessible through the subcostal arch approach [11]. However, the subcostal approach in da Vinci SP surgery offers the advantage of a free lateral thoracic site, thus allowing the assistant to operate from the lateral thoracic area, without fighting from the robotic body. This structural advantage makes it possible to perform quick and safe handling in emergencies, which is a significant benefit. While the presence of dense apical adhesions posed challenges for initial lung mobilization, our experience indicates that the da Vinci SP system did not compromise adequate lymph node dissection in the superior aspects of the chest. Of the 21 patients who underwent mediastinal lymph node dissection in the SP group, 11 underwent upper mediastinal dissection (2R and 4R), and there were no instances where mediastinal lymph node dissection could not be completed due to structural limitations of the da Vinci SP arms. Therefore, while the da Vinci SP enables essential procedures for anatomical lung resection, particularly via the subcostal approach, its limitations—such as the absence of integrated energy devices and staplers—suggest that it cannot fully replace the multi‐port da Vinci Xi, especially in complex cases requiring extensive dissection or additional ports. To cover the full spectrum of thoracic surgeries, including advanced cases or those with severe adhesions, maintaining both platforms may be necessary in institutions that perform a wide range of robotic procedures. Therefore, although technically feasible for less complex cases, the benefits must be weighed against the unique setup and assistant requirements of the da Vinci SP. For 'less complex' cases, the da Vinci Xi or even conventional VATS might still be efficient options, and the da Vinci SP's niche may be more defined by its single‐incision, chest incision‐free advantages where applicable and beneficial to the patient.

Regarding postoperative complications, there were no significant differences in the incidence of Grade II or higher complications according to the Clavien‐Dindo classification between the da Vinci SP and da Vinci Xi groups. However, the postoperative complication rate in the da Vinci SP group was relatively low at 6.7%, which is comparable to the previously reported rate of 8% [11]. The advantages of the subcostal arch approach include the preservation of intercostal nerves, the ease of large specimen retrieval, and the preservation of the intercostal muscles, which can help preserve the postoperative respiratory function and reduce pain [11, 13]. In contrast, the postoperative complication rate in the da Vinci Xi group was 30%. A meta‐analysis comparing the incidence of postoperative complications after anatomic resection for non‐small cell lung cancer between RATS and VATS showed that the complication rate ranged from 26.7% to 36.1% in the RATS group and from 26.1% to 41.4% in the VATS group [14, 15, 16]. This indicates that the postoperative complication rate for RATS is comparable to that of VATS, and our study results align with this trend. Therefore, the postoperative complication rate of da Vinci Xi is consistent with previous reports, with no specific concerns regarding proficiency or the perioperative outcomes. Furthermore, no complications specific to the SP were observed. However, we considered it necessary to be cautious of postoperative bleeding due to diaphragmatic injury, organ damage due to peritoneal injury, and intraoperative cardiac failure due to cardiac compression in the left pleural cavity.

The limitations of this study include its single‐center retrospective design, which may limit the generalizability of the results to other institutions or healthcare settings. Additionally, only one surgeon performed the da Vinci SP procedures, which may have introduced some bias related to the surgeon's experience of the da Vinci Xi and learning curve with the new technology. The absence of locally advanced cases in the da Vinci SP group also limited the applicability of the findings to more complex cases of lung cancer. Furthermore, the small sample size of the da Vinci SP group may have limited the statistical power, and the follow‐up period neglected to include long‐term postoperative outcomes and complications. Additionally, the case selection was uneven, with a higher proportion of segmentectomies and more complex cases (e.g., bilobectomy with bronchoplasty and chest wall resection) performed in the da Vinci Xi group, which may have confounded the outcome. Lastly, the retrospective nature of the study makes it prone to selection bias and potential inaccuracies in the data collection.

In conclusion, the da Vinci SP can be safely introduced for anatomic lung resection, with initial outcomes comparable to those of established multi‐port RATS. While the observed postoperative complication rate in the da Vinci SP group was relatively low, further accumulation of cases, particularly more complex ones, is needed to definitively compare complication rates with the da Vinci Xi group, given the initial case selection differences. Although a learning curve exists, surgeons experienced with the da Vinci Xi platform can transition safely to the da Vinci SP, achieving comparable initial outcomes. Furthermore, da Vinci SP has the potential to reduce postoperative complications, representing a promising evolution in minimally invasive thoracic surgery.

## Author Contributions

**Teruhisa Kawaguchi:** conceptualization, data curation, formal analysis, writing – original draft. **Fumiaki Watanabe:** data curation, writing – review and editing. **Shinji Kaneda:** data curation, writing – review and editing. **Daisuke Ito:** data curation, writing – review and editing. **Koji Kawaguchi:** conceptualization, data curation, project administration, supervision, writing – review and editing.

## Funding

The authors have nothing to report.

## Consent

Informed consent was obtained from research participants via an opt‐out method.

## Conflicts of Interest

The authors declare no conflicts of interest.

## Data Availability

The data underlying this article will be shared on reasonable request to the corresponding author.
